# Cue Reactivity Is Associated with Duration and Severity of Alcohol Dependence: An fMRI Study

**DOI:** 10.1371/journal.pone.0084560

**Published:** 2014-01-06

**Authors:** Zsuzsika Sjoerds, Wim van den Brink, Aartjan T. F. Beekman, Brenda W. J. H. Penninx, Dick J. Veltman

**Affiliations:** 1 Department of Psychiatry, Neuroscience Campus Amsterdam, VU University Medical Center, Amsterdam, The Netherlands; 2 Department of Psychiatry, Academic Medical Center, University of Amsterdam, Amsterdam, The Netherlands; 3 Department of Psychiatry, Leiden University Medical Center, Leiden, The Netherlands; 4 Department of Psychiatry, University Medical Center of Groningen, Groningen, The Netherlands; Institute of Psychiatry at the Federal University of Rio de Janeiro, Brazil

## Abstract

**Introduction:**

With the progression of substance dependence, drug cue-related brain activation is thought to shift from motivational towards habit pathways. However, a direct association between cue-induced brain activation and dependence duration has not yet been shown. We therefore examined the relationship between alcohol cue-reactivity in the brain, cue-induced subjective craving and alcohol dependence duration and severity. Since alcohol dependence is highly comorbid with depression/anxiety, which may modulate brain responses to alcohol cues, we also examined the relation between comorbid depression/anxiety and cue-reactivity.

**Methods:**

We compared 30 alcohol dependent patients with 15 healthy controls and 15 depression/anxiety patients during a visual alcohol cue-reactivity task using functional magnetic resonance imaging blood oxygenated level-dependent responses and subjective craving as outcomes. Within the alcohol dependent group we correlated cue-reactivity with alcohol dependence severity and duration, with cue-induced craving and with depression/anxiety levels.

**Results:**

Alcohol dependent patients showed greater cue-reactivity in motivational brain pathways and stronger subjective craving than depression/anxiety patients and healthy controls. Depression/anxiety was not associated with cue-reactivity, but depression severity in alcohol dependent patients was positively associated with craving. Within alcohol dependence, longer duration of alcohol dependence was associated with stronger cue-related activation of the posterior putamen, a structure involved in habits, whereas higher alcohol dependence severity was associated with lower cue-reactivity in the anterior putamen, an area implicated in goal-directed behavior preceding habit formation.

**Conclusion:**

Cue-reactivity in alcohol dependence is not modulated by comorbid depression or anxiety. More importantly, the current data confirm the hypothesis of a ventral to dorsal striatal shift of learning processes with longer dependence duration, which could underlie increasingly habitual substance use with progressing substance dependence.

## Introduction

Substance use disorders like alcohol dependence (AD) are considered chronic, relapsing brain disorders [1], [2], characterized by compulsive drug use despite negative consequences [3], [4]. The development of drug dependence is thought to follow a gradual transition during which initial hedonic effects of drugs decrease [4], and the gradual formation of drug habits contributes to the compulsive character of dependence [5], leading to relapse even after longer periods of abstinence. According to the incentive salience theory [6], repeated drug exposure renders cues to become associated with drug use through Pavlovian conditioning, resulting in an increased salience of drugs and drug-related stimuli, which initiates drug-related responses such as craving [7]. Consequently, the presence of drug-related cues alone can act as a conditioned reinforcer and precipitate instrumental drug-seeking and drug-using behavior, also described as Pavlovian-instrumental transfer [8]. Through this mechanism, cue-elicited craving is implicated in theories of relapse [9], and greater insight into its neurobiological substrate could improve treatments.

Imaging studies in human alcohol abusers have shown alcohol cue-induced activation of brain areas involved in motivational pathways, including orbitofrontal cortex (OFC), medial prefrontal cortex (MPFC) and adjacent anterior cingulate cortex (ACC) and ventral striatum (VS) (for a review see [10]). These areas have shown reactivity to natural rewards such as food and sex in healthy samples [11], and to high-calorie food in obese individuals [12], suggesting a more generalizable involvement of these areas in motivational drives. However, the formation of drug habits following prolonged drug use is thought to be associated with further neural adaptations over time. In rodent studies, prolonged drug use results in a shift in brain involvement from the ventral to the dorsomedial and dorsolateral striatum through a dopamine-dependent cascading loop between sub-regions of the striatum [13], [14]. Similarly, in humans, a study in very experienced crack cocaine users (mean duration 11 years) showed strong activation in the dorsal striatum in response to cocaine cues [15]. PET studies in cocaine abusers show that this dorsal striatal activation is associated with significantly increased dopamine levels in the dorsal striatum, which is in turn positively associated with cocaine craving [16]–[18]. However, these studies did not provide information on a possible time-dependent gradual shift away from reward-related towards more habit driven drug use and its neural correlates. This knowledge is until now almost exclusively based on pre-clinical animal studies [5], and there is currently only one study in humans that has tried to address this issue [19]. In accordance with the hypothesis of a shift of learning processes from ventral to dorsal parts of the striatum with progressing alcohol dependence, this study showed that light social drinkers had higher cue-induced fMRI activations in the ventral striatum (VS) compared with heavy – mostly alcohol dependent – drinkers, whereas heavy drinkers showed significantly higher activations compared with light drinkers in the dorsal striatum (DS). Moreover, VS activation in this study was negatively correlated with a self-report measure of compulsive drinking, whereas DS activation showed a positive correlation with this measure, indicating a shift from VS to DS cue-induced activation when alcohol use becomes compulsive. However, a direct association with the duration of alcohol dependence was not made in this study. Of note, a recent coordinate-based meta-analysis, using activation likelihood estimation [20], analyzed 28 alcohol cue-reactivity imaging studies and found that reactivity to alcohol cues (visual, odor, taste) in the VS was typically reported in heavy alcohol users and less so in individuals with an established diagnosis of alcohol dependence. This further corroborates the hypothesis that the VS is predominantly involved during early stages of (problematic) alcohol use, whereas with a longer drinking history, cue-reactivity shifts towards the DS.

However, the association between the duration of alcohol dependence and cue-reactivity has, to the best of our knowledge, not been examined yet, and the influence of Pavlovian cues on the neurobiological processes towards habitual drug use in humans still needs to be elucidated, especially in relation to addiction characteristics such as duration and severity of dependence. Therefore, the current study aimed to investigate the association between alcohol cue-reactivity during fMRI scanning, craving and alcohol dependence characteristics such as duration and severity. Within a large sample of AD patients we focused on striatal activation and we hypothesized to observe larger involvement of posterior and dorsolateral striatal areas at the expense of VS involvement with a longer history of AD, indicating a cue-induced shift from reward pathways to habit pathways with longer AD duration.

An often overlooked characteristic of alcohol dependence is the high comorbidity with depression/anxiety disorders [21]. Most neurobiological studies in AD, however, either exclude AD patients with comorbid depressive and/or anxiety disorders, thus collecting data on a very specific sample of limited clinical relevance, or fail to consider the presence of comorbid depression and/or anxiety as a potential confounder. This is important, because depression/anxiety disorders are often characterized by anhedonia with reduced brain reward responses towards external, potentially rewarding cues compared with healthy subjects [22], [23]. In order to account for this complexity, we included a clinically relevant group of AD patients without excluding AD patients with comorbid depression/anxiety disorders and studied the effect of depression/anxiety within the AD group on cue-reactivity. In addition, we added two comparison groups: a group of healthy controls (HC) and a group of patients with depression/anxiety (D/A). We hypothesized stronger regional brain activation and craving in reaction to alcohol-related cues in AD patients compared with both D/A patients and HCs, an effect that would be mainly associated with alcohol dependence characteristics such as duration and severity and not, or only weakly, with depression/anxiety severity. In addition, we expected reduced regional brain activation in reaction to alcohol-related cues in D/A patients compared with HCs.

## Materials and Methods

### Participants

Forty-two patients were included with a current (<6 months) DSM-IV diagnosis of AD and no comorbid lifetime Axis-I diagnosis other than major depressive disorder (MDD) and/or anxiety disorders. Twenty-two patients with a current (<6 months) DSM-IV diagnosis of major depression and/or anxiety disorder (generalized anxiety disorder, panic disorder, social anxiety disorder), but no other lifetime diagnosis of Axis-I disorders were included as a psychiatric comparison group (D/A). A group of 21 individuals without any lifetime psychiatric disorder was included as a healthy control group (HC). Both comparison groups were matched on age, gender and education with the AD group.

In order to study a heterogeneous sample of AD subjects with regard to the duration and severity of the disorder, the AD group was recruited from two sources. One half of the AD group was recruited from local addiction treatment clinics; the other half of the AD group was recruited from the Netherlands Study of Depression and Anxiety (NESDA) [24], a large multi-site naturalistic cohort study including patients from primary care and outpatient mental health services. The D/A and HC groups were also recruited from NESDA.

After data collection was completed, we excluded the following participants: subjects with: a) missing data (3 AD, 2 D/A, 1 HC); b) imaging data of poor quality (4 AD, 3 D/A, 2 HC); c) excessive movement during scanning (1 AD); d) not-earlier reported coma (1 HC); or e) testing positive for cocaine or benzodiazepines (5AD, 2D/A, 0 HC). In addition, two HC subjects with substantial alcohol problems (AUDIT score >10) were excluded. The final dataset included 30 AD, 15 D/A, and 15 HC participants for data analyses.

### Ethics Statement

The research protocol was approved by the medical ethical review board of the participating universities, and conducted according to the principles expressed in the Declaration of Helsinki. All participants provided written informed consent. All potential participants who declined to participate or otherwise did not participate were eligible for treatment (if applicable) and were not disadvantaged in any other way by not participating in the study.

### Procedure and clinical assessments

Participants were screened for current and lifetime psychiatric diagnosis using the Composite International Diagnostic Interview (CIDI, version 2.1) [25]. All participants were free of major internal or neurologic disorders and MRI contraindications, did not use psychotropic medication other than a stable prescription of selective serotonin reuptake inhibitors or infrequent benzodiazepine use, and were free of current substance abuse or dependence, other than alcohol for the AD group, and smoking for all three groups. All participants were asked to abstain from alcohol 24 hours and from caffeine a few hours prior to the study and had a confirmed breath alcohol level of 0.00% (Alcoscan Daisy-AL7000). Mean abstinence duration in the AD group was 12 days (see Table 1). No participants scored higher than 8 points on the withdrawal symptom-checklist CIWA-Ar, [26] and were therefore considered withdrawal-free.

**Table 1:** Sample Characteristics.

	Alcohol Dependent patients N = 30	Depression/Anxiety patients N = 15	Healthy Controls N = 15			
	Mean (SD)	Mean (SD)	Mean (SD)	*test*	*test-statistic*	*p-value*
*Demographics*						
Age	46.5 (8.5)	48.4 (10.6)	46.8 (10)	*H*	*0.94*	*0.626*
Males, N (%)	16 (53.3)	6 (40)	11 (73.3)	*X^2^*	*3.43*	*0.18*
Right handed, N (%)	27 (90)	14 (93.3)	12 (80)	*X^2^*	*1.46*	*0.483*
Years of education	13.1 (3.5)	13.3 (3.9)	14.1 (3.6)	*H*	*1.02*	*0.6*
*Clinical characteristics*						
Depression severity (IDS score)	20.5 (10.2)	18.8 (12.4)	3.1 (3.9)	*F*	*17.18*	*<0.001*
Anxiety severity (BAI score)	11.8 (10.2)	11.1 (9.2)	1.5 (2.6)	*H*	*21.6*	*<0.001*
AD severity (AUDIT score)	18.1 (8)	1.5 (1.4)	3.7 (2.1)	*H*	*40.32*	*<0.001*
Drinks per drinking day	3.1 (1.4)	0.9 (0.7)	1.3 (0.7)	*H*	*29.56*	*<0.001*
Drinking days per week	4.4 (2.9)	0.6 (0.8)	2.7 (2.5)	*H*	*19.11*	*<0.001*
Drinks per week	15.4 (12.5)	0.8 (1.0)	4.4 (4.6)	*H*	*24.15*	*<0.001*
Duration of AD (years)	15 (11.8)	-	-		-	-
Abstinence (days; minimum 24 hrs.)	12.2 (20.4)	31.3 (78.4)	6.8 (16.4)	*H*	*2.74*	*0.254*
Smokers, N (%)	17 (56.7)	5 (33.3)	0 (0)	*X^2^*	*13.92*	*0.001*
*Behavioral characteristics*						
Craving Before	31 (13.6)	17.9 (5.8)	19.3 (6.2)	*F*	*10.16*	*<0.001*
Craving After	34.5 (16.7)	18.6 (6.7)	21.6 (9.6)			
Mean Valence score	4.7 (1.4)	2.6 (1.5)	3.9 (1.5)	*H*	*15.29*	*<0.001*

Abbreviations: AD, alcohol dependants; AUDIT, Alcohol Use Disorder Identification Test; BAI, Beck Anxiety Inventory; HC, healthy controls; IDS, Inventory of Depressive Symptomatology; M, mean; N, number; SD, standard deviation; *F*, One-way ANOVA; *H*, Non-parametric Kruskall Wallis test; *X^2^*, Chi-square test.

For sample-description purposes, self-report questionnaires were filled out to assess demographic and clinical information. Handedness was measured by the Hand Preference Questionnaire [27]. The presence and severity of alcohol use disorders and nicotine dependence was assessed by the Alcohol Use Disorder Identification test (AUDIT; [28], and the Fagerstrom Test for Nicotine Dependence [29]. AD duration in years was calculated by subtracting the age of onset as assessed by the CIDI from the age during scanning. Anxiety severity was assessed by the Beck Anxiety Inventory (BAI) [30] and depression severity by the Inventory of Depressive Symptomatology (IDS) [31].

The Desire for Alcohol Questionnaire (DAQ) [32], a 14-item self-report questionnaire measuring craving for the use of alcohol, was assessed immediately before and after scanning to measure baseline craving and cue-induced subjective craving, respectively. A craving difference score was calculated by subtracting the craving score before scanning from the craving score after scanning. A positive difference score indicated an increase in craving after alcohol cue presentation during scanning.

### Cue Reactivity Paradigm

To measure cue-induced brain activity, an event-related visual cue-reactivity fMRI task was used, during which 50 alcohol-related pictures and 50 graphically matched affectively neutral pictures were presented to all participants. A large part of the alcohol stimuli were taken from alcohol databases also used by Wrase *et al* (2002) and Grüsser *et al* (2000) and supplemented with alcohol-related pictures from the web, after validation on positive valence by volunteers with alcohol use disorders. Neutral pictures were taken from the International Affective Picture System (IAPS) (Lang *et al*, 2008), after matching for graphic characteristics with the chosen alcohol cues. Participants were asked to watch and attend to the stimuli. To ensure maintained attention, participants were instructed to press a button when a face was shown on the picture. Of every 50 pictures in each set, 10 contained pictures with faces, which were modeled as a regressor of no interest, leaving 40 alcohol and 40 neutral pictures for analysis. Each picture was presented for 5 seconds, with an interstimulus interval jittered between 500 and 1000 ms. To promote cue-induced craving, the pictures were presented in random order in blocks of various lengths (4-6 pictures), neutral and alcohol blocks alternating. Imaging analyses were performed in an event-related fashion. Every block was preceded by a fixation cross (jittered 4 to 6 seconds), which was used as an implicit baseline. All participants were asked to rate the alcohol pictures on positive valence for drinking on a scale from 0 (no valence) to 9 (very salient) immediately after scanning.

### Imaging Data Acquisition & Preprocessing

Magnetic resonance imaging was performed at the Academic Medical Centre (AMC) in Amsterdam using a 3T Philips Intera full-body MR system (Philips Medical Systems, Best, the Netherlands) with a phased array SENSE RF 8-channel head coil. Functional blood oxygen level-dependent (BOLD) signals were sequentially acquired with a T2*-weighted, gradient-echo planar imaging (EPI) sequence and an estimated time-course series of approximately 290 volumes (self-paced) per session (TR/TE = 2300 ms/20 ms; matrix size = 96×95, voxel size = 2.29×2.29×2.50 mm; slices = 45). To minimize susceptibility and distortion artifacts in OFC each volume was scanned with an orientation of 30° from the Anterior-Posterior Commissure (AC-PC) line [33]. Three-dimensional T1-weighted images were obtained using a gradient echo sequence for anatomical reference with the EPI data (TR/TE = 9 ms/3.6 ms; matrix size = 256×231; voxel size = 1×1×1 mm; slices = 170).

Imaging data were preprocessed and analyzed using SPM8 (Wellcome Trust Centre for Neuroimaging, London, UK). Images were manually reoriented to the AC-PC line, slicetimed, realigned, warped to Montreal Neurological Institute (MNI) space, and smoothed using a Gaussian kernel of 8 mm at full-width-at-half-maximum.

### Statistica*l* analyses

Sample characteristics and behavioral data were analyzed using IBM SPSS Statistics 20 (IBM, New York, NY, USA). Demographic and clinical characteristics were compared between groups using one-way ANOVA procedures, and non-parametric Kruskal-Wallis or Chi-square tests depending on the nature and the distribution of the variables. Craving before and after fMRI scanning was analyzed using repeated measures group (AD, D/A, HC) by session (before and after scanning) ANOVA, and change scores were tested within-groups against a value of 0 (no change) by means of a Student's *t*-test. Follow-up tests were conducted to evaluate pairwise differences among the three groups, controlling for Type-I error across tests by using the Bonferroni approach. Correlation analyses between behavioral parameters, and alcohol use and other clinical characteristics were performed using Spearman's Rho. Significance was set to *P*<.05.

Statistical analysis of individual imaging data was performed using a first-level fixed effects analysis, in the context of the General Linear Model [34], with the onsets of every picture or baseline cross convolved with a canonical hemodynamic response function to model each outcome type. To remove low-frequency signal drift, a high-pass filter (128 Hz) was applied. Two main trial types were distinguished, i.e. alcohol and neutral stimuli, whereas pictures requiring a button press were modeled as a regressor of no interest. For each subject a contrast image Alcohol>Neutral was constructed to examine regional brain activation related to alcohol cue-reactivity. These contrast images were entered into a second level random-effects analysis using a one-way ANOVA design to investigate between-group effects. In addition, we checked for group effects on the Neutral>Baseline contrast, because we previously found that substance dependent patients may show a stronger brain response to neutral compared to baseline pictures [35]. Within the AD group, whole-brain linear regression analyses were performed to study associations between alcohol-cue induced brain activation and duration and severity of AD, and subjective craving. Additionally, we checked for effects of smoking and depression/anxiety severity on cue-induced brain activity by including the amount of smoked cigarettes per day, and IDS and BAI scores in separate regression analyses within the AD group.

To examine the hypothesized striatal shift during cue-reactivity, we performed *a priori* defined region of interest (ROI) analyses in the striatum. Bilateral masks for the putamen, caudate nucleus and globus pallidus were derived from the automatic anatomical labeling (AAL) atlas [36] incorporated in the WFU-PickAtlas Tool v2.5.2 [37]. The WFU-Pickatlas does not provide an anatomical mask for the most ventral part of the striatum, comprising the nucleus accumbens. Therefore we selected a bilateral VS mask [38] defined in the BrainMap database [39], and created a binary mask from the probabilistic mask at *P*>.70 using the ImCalc-tool in SPM8. Following methods in the recent study by Vollstadt-Klein and colleagues [19], this VS mask was subtracted from the AAL-defined striatal areas to distinguish between ventral and dorsal striatal areas. For a visualization of the ROIs, please see Figure S1. Recently, studies focusing on the transfer towards habit-related behavior in healthy human participants indicate that within the putamen an additional distinction can be made between the anterior and posterior putamen [40], [41], which may correspond to the dorsomedial versus dorsolateral striatal involvement seen in rodents. Because this distinction is primarily implicated in instrumental rather than Pavlovian conditioning, we did not add this distinction to the statistical ROI analyses, but to explore any (dorsal) putamen findings, we used the anterior vs. posterior demarcation at MNI-coordinate y = 2 proposed earlier [40]. We report significant brain activations within the striatal areas at *P*<.05 that survived family-wise error (FWE) correction for multiple comparisons on the voxel-level within the ROIs using a small volume correction (*P*_SVC-FWE_<.05) [42]. Additionally, for explorative reasons we report whole-brain imaging results in the supplementary material at *P*<.005 uncorrected, and to further protect against type-I error, a voxel extent threshold of 15 voxels [43].

## Results

### Sample characteristics

As expected, groups differed on alcohol use characteristics (*H*_2,60_ = 40.32; *P*<.001), because the AD group scored significantly higher than the HC and D/A groups (both *P*<.001) whereas the HC and D/A groups did not differ from each other (*P* = .919). The AD and the D/A group reported more depressive and anxiety symptoms than the HC group (*F*_2,57_ = 17.175; *P*<.001, pairwise both *P*<.001; and *H*_2,60_ = 21.598; *P*<.001, pairwise both *P*<.001, respectively). The AD and the D/A group did not differ significantly on depression or anxiety severity (pairwise, *P* = 1.000 and *P* = .918, respectively). The groups differed in the percentage of smokers (*χ^2^*_2,60_ = 13.923; *P*<.001) and number of cigarettes smoked (*H*_2,60_ = 14,472; *P*<.001): none of the HC participants smoked, whereas 17 of the 31 (57%) AD patients and 5 of the 18 (33%) D/A participants did. The number of smoked cigarettes per day, however, did not show associations with other demographic and clinical sample characteristics.

Groups differed in valence ratings (H_2,59_ = 15.292; *P*<.001), indicating that AD patients found the alcohol pictures more positively influencing their desire to drink than the D/A group (pairwise, *P*<.001), whereas the AD and the D/A group did not differ from HC (pairwise, *P* = .294 and *P* = .153, respectively).

The mean duration of alcohol dependence in AD patients was 15.0 years with a large variation (SD  = 11.8 years), suggesting substantial heterogeneity. Duration of AD was positively associated with alcohol use severity (*r* = .441; *P* = .015).

For detailed information on sample characteristics, see Table 1.

### Self-reported craving

**Figure 1:**
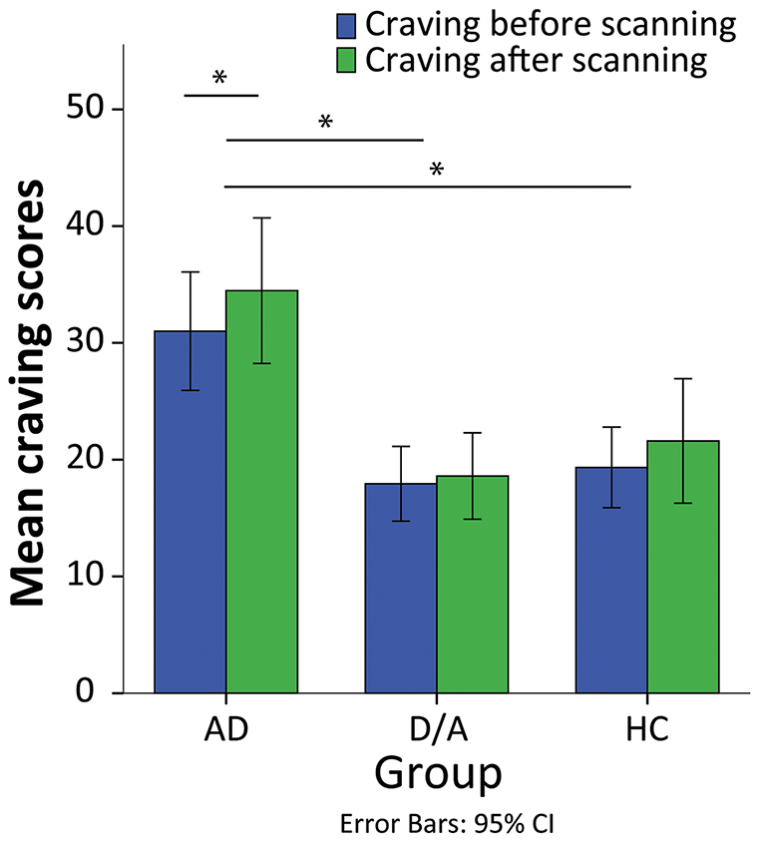
Mean craving scores before and after scanning in the three groups. The AD group showed higher craving scores compared to the D/A (*P* = .001) and HC (*P* = .003) groups. The difference between before and after was only significant for the AD group (*P* = .004). Abbreviations: AD, alcohol dependents; CI, confidence interval; HC, healthy controls; D/A, psychopathology controls.

Repeated measures ANOVA showed a main effect of group (*F*_2,57_ = 10.158; *P*<.001): the AD group scored significantly higher than the D/A and HC groups both at baseline and after cue-exposure (AD vs. D/A: *P*<.001; AD vs. HC: *P<.*001). A main effect of session showed increased craving after scanning (*F*_1,57_ = 6.260; *P = .015*). No group*session interaction was seen. Within-group one-sample t-tests indicated that the AD group showed significantly increased craving after fMRI scanning (M = 3.3; *P* = .004), whereas this difference in craving before and after the scanning session was not significant for the D/A group (*P* = .682) or the HC group (*P* = .167) (Fig. 1).

Subjective craving scores correlated positively with alcohol severity according to the AUDIT (before scanning: r = .465; *P*<.001; after scanning: r = .494; *P*<.001). Subjective craving scores also correlated positively with depression severity in the total sample (before scanning: r = .439; *P*<.001; after scanning: r = .334; *P*<.001), in the AD group (before scanning: r = .523; *P* = .003; after scanning: r = .403; *P* = .027), and in the HC group (after scanning only: r = .588; *P* = .021).

There were no associations between the number of smoked cigarettes per day and alcohol craving scores, neither in the AD nor in the D/A group.

### Imaging data

#### Group comparisons

The fMRI cue-reactivity paradigm activated a wide network of brain areas implicated in the motivational and reward system, as well as attention and object recognition networks, replicating studies on alcohol cue-reactivity [44], [45]. Main effects of Alcohol>Neutral pictures over the whole sample and per group are shown in Table S1.

**Figure 2:**
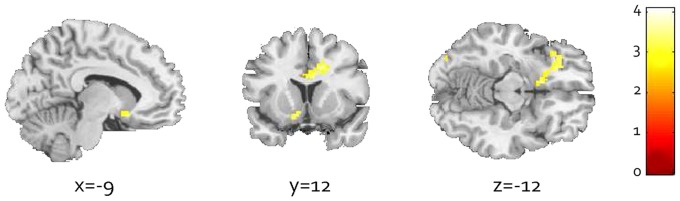
Cue-reactivity in AD patients compared with both comparison groups (D/A patients and HC). Two clusters are visible: Activation of a large cluster reaching from the OFC to the VS, and in the ACC**.** Colored bar: *Z*-scores from 0 to 4, *P*<0.005, clustersize threshold > = 15 voxels.

ANOVA group comparisons on Alcohol>Neutral pictures showed higher cue-induced brain activation in the AD group compared to the D/A and HC groups (See Table S2). Increased VS activation in the comparison between AD and D/A patients approached significance (*P*_FWE_<.1). Given the similar activation patterns in the two comparison groups (15 D/A, 15 HC), we performed a two-sample t-test comparing 30 AD patients with 30 comparison subjects (15 D/A +15 HC subjects) to further investigate cue-reactivity in AD while increasing statistical power. In AD patients (N = 30) compared with the pooled comparison groups (N = 30), activation of the VS survived ROI FWE-correction (*P*_FWE_<.05) (see Figure 2). Additionally, during whole-brain exploration of group comparisons at a more liberal threshold (P<0.005 whole-brain uncorrected, cluster extent  = 15 voxels), the AD group showed larger activations in previously reported cue-reactivity areas like the ACC, medial PFC, IFG, OFC, VMPFC, caudate body, thalamus and globus pallidus (see Table S2).

The AD group did not show lower activation compared with the two control groups.

Checking for confounding group effects on neutral pictures versus baseline trials only yielded expected results in bilateral occipital cortices (*Z* = 3.64), right hippocampal formation (*Z* = 3.58), right inferior parietal lobule (*Z* = 3.63), and bilateral dorsolateral (*Z* = 3.56) and dorsomedial (*Z* = 3.38) prefrontal cortices, and no effects in ventral prefrontal and striatal areas.

#### Regression analyses within the AD group

**Figure 3:**
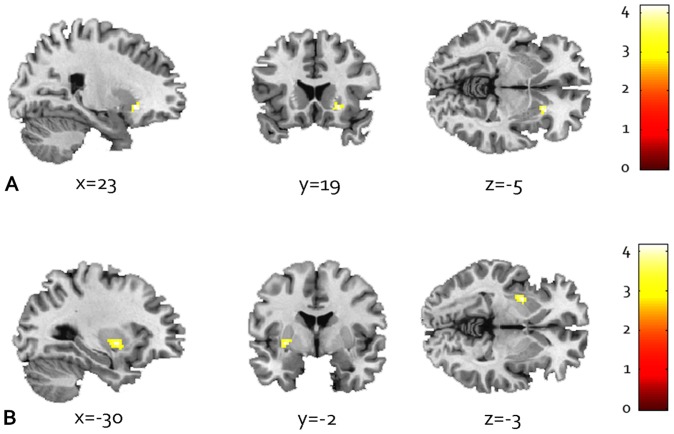
Regression analyses within AD group. a. A negative association between alcohol use disorder severity (AUDIT score) and cue-reactivity in the anterior putamen (*Z* = 3.79; *P*_FWE_<.05). b. A positive association between alcohol cue-reactivity and duration of alcohol dependence shows activation in the posterior putamen (*Z* = 3.64; *P*_FWE_<.05). Displayed at *P*<0.005 uncorrected; cluster size threshold > = 15 voxels, masked with bilateral putamen.

Within the AD group, whole-brain correlation analysis showed no positive correlation between alcohol use disorder severity (AUDIT) and alcohol cue-reactivity in the a priori regions of interest, but a negative association between alcohol use disorder severity and activation of the right anterior putamen after small volume FWE-correction was found (*P*_SVC-FWE_<.05) (Z = 3.79) (see Table S3 and Figure 3b).

AD duration showed a positive association with alcohol cue-reactivity in several areas across the brain when exploring the whole-brain (see Table S3), with a significant association in left posterior putamen (Z = 3.64; *P*_SVC-FWE_<.05). Since AD duration was correlated with age (*ρ* = .444; *P*<.014) we *post-hoc* performed multiple regression analyses in SPM, revealing that posterior putamen activation was correlated with AD duration, but not with age.

Self-reported cue-induced craving scores showed a weak association with alcohol cue-reactivity during whole-brain exploration; however no craving related activation was seen in the striatum.

Depression and anxiety severity within the AD group showed no or only a weak positive association with alcohol cue-reactivity, and a negative association in areas of no a-priori interest during whole-brain explorations. No areas survived small-volume FWE-correction.

Post-hoc analysis showed that the amount of cigarettes was not associated with cue-reactivity in areas seen in the previous analyses or within the *a-priori* ROIs.

For details on the activations of the regression analyses in the a-priori set regions of interest, and additional explorative whole-brain activations at a more liberal threshold see Table S3.

## Discussion

In the current study, AD patients showed higher baseline and cue-induced craving than HCs and D/A patients. The increase in craving after alcohol cue-exposure compared with baseline craving before cue-exposure was present in the AD group but not in the two comparison groups, indicating that subjective craving increased disproportionately in AD patients after visual alcohol-cue exposure. In addition, our imaging results showed that exposure to visual alcohol cues during MRI-scanning increased brain activation in AD patients compared with D/A patients and HCs in brain areas that are part of the motivational network, such as the OFC, VS and MPFC.

These findings confirm those of numerous previous studies on cue-reactivity comparing heavy substance users with healthy controls [10], [46]. It is hypothesized, however, that motivational pathways such as the VS are mainly involved in the earlier-stage of problematic drug use [20], and that with prolonged drug use a transition towards habit pathways, comprising dorsolateral parts of the striatum, is expected [47]. For example, pharmacological studies have shown that blocking the rewarding effects of drugs by administrating dopamine D2 blockers or the mu-opiate antagonist naltrexone is only successful in non-dependent heavy alcohol users, or ‘early-stage’ alcohol dependents [4], when rewarding effects of drug intake and the motivational pathways such as the VS still guide alcohol use. Therefore, VS activation would mainly be expected in these ‘early stage’ AD patients. Our large sample of alcohol dependents comprised a heterogeneous group regarding severity and duration of AD, with about half of the AD patients currently not in treatment, and with less severe and shorter AD. Therefore, the observed effect of cue-exposure on VS activation could be driven by the relatively large group of less chronic AD patients. However, a regression analysis on the possible influence of severity and duration of AD on cue-induced VS activation did not confirm this hypothesis. These analyses did show that with longer lasting AD, the involvement of the posterior putamen during alcohol cue-exposure is more pronounced. Posterior putamen has previously been shown to be involved in habitual behavior [40], [41], [48], and the current result indicates that, following Pavlovian cues, more chronic AD patients activate habit pathways, which confirms the habit formation theory [5]. Finally, we found lower activation in the anterior putamen, implicated in goal-directed behavior, with higher AD severity, which in turn was positively correlated with AD duration. This finding suggests that the shift from motivational or goal-directed pathways towards pathways involved in habit formation thought to be time dependent (progression over time) is partly mediated by severity of AD. However, future studies using prospective designs should elucidate on the shift away from motivational and goal-directed pathways in favor of increasing involvement of habit pathways as a function of dependence duration as well as severity.

A direct association between craving scores and cue-reactivity in motivational or habitual brain pathways in AD patients was not clearly present. This could be due to a mismatch between self-reported subjective craving and the objective method to assess cue-conditioned brain responses to alcohol-related stimuli. Subjective craving is difficult to measure in human subjects [49] due to denial by detoxified alcohol-dependent patients, despite high relapse rates [9]. Our observation of a lack of association between cue-reactivity and craving, however, is in line with the hypothesis that drug cues can motivate drug intake even in the absence of conscious craving, because habitual drug use has become involved in the maintenance of drug dependence, rather than craving [9]. This could also explain the lack of association between self-reported craving and duration of alcohol dependence in this study.

An additional aim of this study was to examine the effect of depression/anxiety on alcohol cue-reactivity, by directly comparing cue-induced brain activation and subjective craving in AD patients (with and without comorbid depression/anxiety disorders) and D/A patients (without AD), because the latter group is known to show decreased reactivity to rewarding stimuli, a phenomenon also known as reward deficiency or anhedonia [22], [23]. In the current study, the D/A group was characterized by lower drinking levels, lower subjective alcohol craving scores, lower valence scores, and lower brain reactivity to alcohol cues compared with the AD group. However, brain reactivity to alcohol cues in D/A patients did not differ from healthy controls. This does not directly confirm a possible blunting effect of depression/anxiety in reactions to alcohol cues. Among heavy drinkers comorbid depression/anxiety symptoms have previously been found to be positively associated with brain activation in response to alcoholic taste cues [50], explaining the association between stress or negative emotional state and relapse after treatment [51]. However, we did not observe a positive association between depression/anxiety symptoms and brain activation in response to visual alcohol cues within our AD group, suggesting that in alcohol dependent populations the presence of depression/anxiety symptoms has no direct effect on neuronal responses to visual alcohol cues. Together, these findings show that the alcohol cue-induced brain activation in our AD group is not influenced by the presence of comorbid D/A disorders, indicating the presence of a strong effect of AD on alcohol cue-induced brain activation even in an ecologically valid and clinically relevant group of AD patients with a high comorbidity with D/A disorders. It should be noted, however, that depressive symptoms were positively correlated with subjective craving rates overall and within the AD group. This is consistent with the idea that depression-related stress induces craving and consequently subsequent relapse [51]. However, we did not show a corresponding association as seen with self-reported craving between depression/anxiety scores and physiological measurements of brain activation, suggesting lower sensitivity of physiological measurements or a more idiosyncratic influence of depression/anxiety severity on craving.

A possible limitation in the current study is, however, that we did not study general positive versus alcohol-specific salient cues, since we were mainly interested in alcohol cue-reactivity in AD patients. Therefore we were not able to test the specificity of a blunted brain response to alcohol cues in D/A patients. We therefore suggest that future cue-reactivity studies should include both alcohol-related cues and non-alcohol related positive and negative cues.

In conclusion, the current study shows that neural responses to drug cues are subject to a change from activation of brain areas involved motivational and goal-directed processes towards activation of brain areas implicated in chronic drug taking habits with increasing severity and longer duration of AD. This observation presumably underlies the instrumental shift from goal-directed towards habitual drug-taking behavior [5], leading to frequent and highly relapsing substance use despite serious negative consequences.

## Supporting Information

Figure S1 **Regions of interest.** Bilateral masks for the putamen, caudate nucleus and globus pallidus were derived from the automatic anatomical labeling (AAL) atlas (Tzourio-Mazoyer et al. 2002) incorporated in the WFU-PickAtlas Tool v2.5.2 (Maldjian et al. 2003). The WFU-Pickatlas does not provide an anatomical mask for the most ventral part of the striatum, comprising the nucleus accumbens. Therefore we selected a bilateral ventral striatal mask (Nielsen and Hansen 2002) defined in the BrainMap database (Fox and Lancaster 1994), and created a binary mask from the probabilistic mask at *P*>.70 using the ImCalc-tool in SPM8. Following methods in the recent study by Vollstadt-Klein and colleagues (Vollstadt-Klein et al. 2010), this VS mask was subtracted from the AAL-defined striatal areas to distinguish between ventral and dorsal striatal areas. References: **Fox** PT, Lancaster JL (1994). Neuroscience on the net. *Science* 266(5187): 994–996. **Maldjian** JA, Laurienti PJ, Kraft RA, Burdette JH (2003). An automated method for neuroanatomic and cytoarchitectonic atlas-based interrogation of fMRI data sets. *Neuroimage* 19(3): 1233–1239. **Nielsen**, F. A. and Hansen, L. K. (2002). Automatic anatomical labeling of Talairach coordinates and generation of volumes of interest via the BrainMap database (Presented at the 8th International Conference on Functional Mapping of the Human Brain, June 2–6, 2002, Sendai, Japan. Available on CD-Rom.). *Neuroimage* 16 (2). **Tzourio-Mazoyer** N, Landeau B, Papathanassiou D, Crivello F, Etard O, Delcroix N, Mazoyer B, Joliot M (2002). Automated anatomical labeling of activations in SPM using a macroscopic anatomical parcellation of the MNI MRI single-subject brain. *Neuroimage* 15(1): 273–289. **Vollstadt-Klein** S, Wichert S, Rabinstein J, Buhler M, Klein O, Ende G, Hermann D, Mann K (2010). Initial, habitual and compulsive alcohol use is characterized by a shift of cue processing from ventral to dorsal striatum. *Addiction* 105(10): 1741–1749.
(TIF)

Table S1 **Main effects of Alcohol>Neutral pictures.**
(DOCX)

Table S2 **Group comparisons. Activated areas during alcohol-cue reactivity.**
(DOCX)

Table S3 **Regression analyses during alcohol-cue reactivity within the group of Alcohol Dependent patients.**
(DOCX)
