# Supplementary figures and images for: Cue Reactivity Is Associated with Duration and Severity of Alcohol Dependence: An fMRI Study
Source: PLoS One. 2014 Jan 6;9(1):e84560. doi: 10.1371/journal.pone.0084560 (PMC3882248; doi:10.1371/journal.pone.0084560)

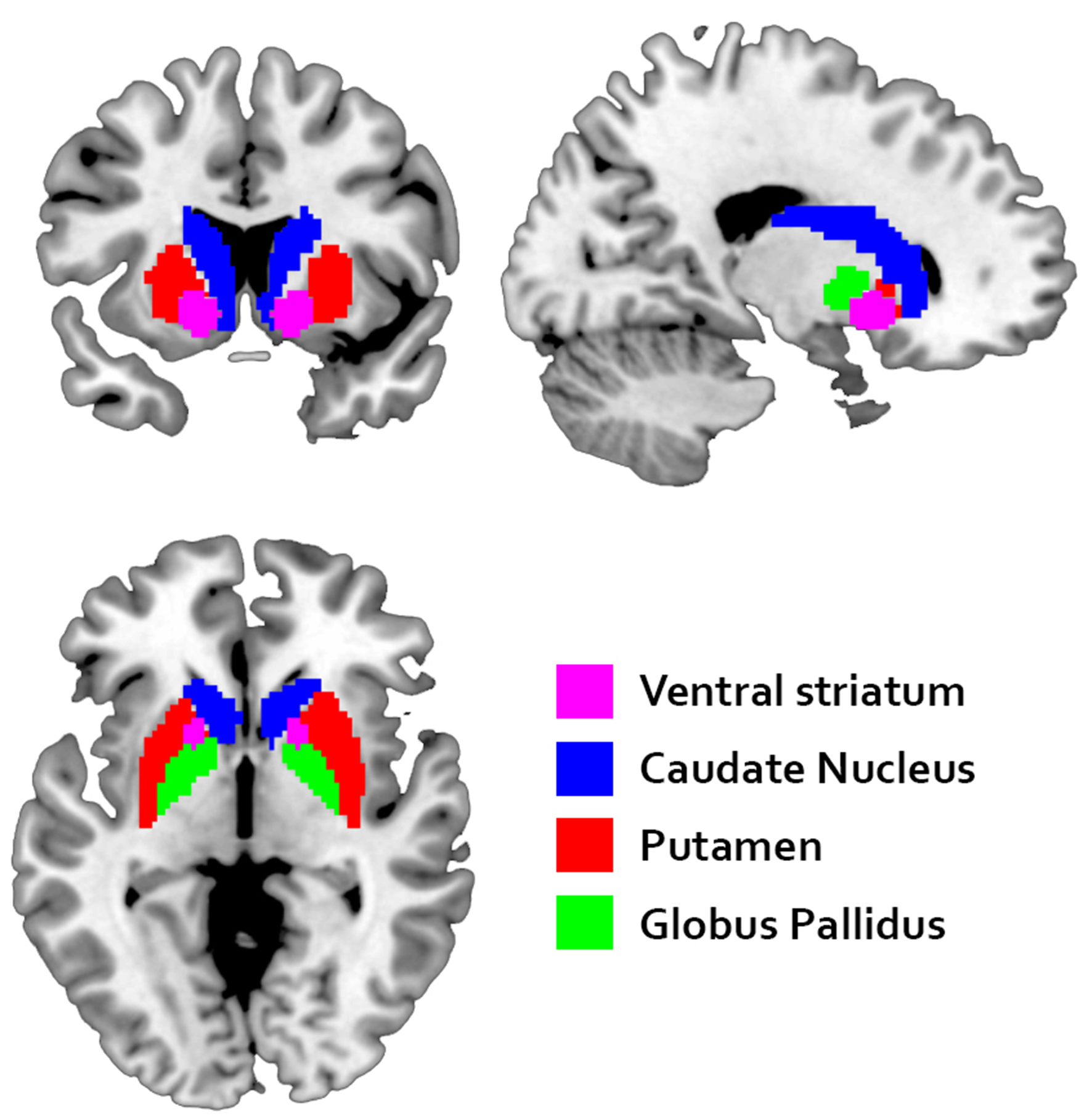

Supplement: Figure S1 — Regions of interest. Bilateral masks for the putamen, caudate nucleus and globus pallidus were derived from the automatic anatomical labeling (AAL) atlas (Tzourio-Mazoyer et al. 2002) incorporated in the WFU-PickAtlas Tool v2.5.2 (Maldjian et al. 2003). The WFU-Pickatlas does not provide an anatomical mask for the most ventral part of the striatum, comprising the nucleus accumbens. Therefore we selected a bilateral ventral striatal mask (Nielsen and Hansen 2002) defined in the BrainMap database (Fox and Lancaster 1994), and created a binary mask from the probabilistic mask at P>.70 using the ImCalc-tool in SPM8. Following methods in the recent study by Vollstadt-Klein and colleagues (Vollstadt-Klein et al. 2010), this VS mask was subtracted from the AAL-defined striatal areas to distinguish between ventral and dorsal striatal areas. References: Fox PT, Lancaster JL (1994). Neuroscience on the net. Science 266(5187): 994–996. Maldjian JA, Laurienti PJ, Kraft RA, Burdette JH (2003). An automated method for neuroanatomic and cytoarchitectonic atlas-based interrogation of fMRI data sets. Neuroimage 19(3): 1233–1239. Nielsen, F. A. and Hansen, L. K. (2002). Automatic anatomical labeling of Talairach coordinates and generation of volumes of interest via the BrainMap database (Presented at the 8th International Conference on Functional Mapping of the Human Brain, June 2–6, 2002, Sendai, Japan. Available on CD-Rom.). Neuroimage 16 (2). Tzourio-Mazoyer N, Landeau B, Papathanassiou D, Crivello F, Etard O, Delcroix N, Mazoyer B, Joliot M (2002). Automated anatomical labeling of activations in SPM using a macroscopic anatomical parcellation of the MNI MRI single-subject brain. Neuroimage 15(1): 273–289. Vollstadt-Klein S, Wichert S, Rabinstein J, Buhler M, Klein O, Ende G, Hermann D, Mann K (2010). Initial, habitual and compulsive alcohol use is characterized by a shift of cue processing from ventral to dorsal striatum. Addiction 105(10): 1741–1749. (TIF) [file pone.0084560.s001.tif]
