# Supplementary material for: Cue Reactivity Is Associated with Duration and Severity of Alcohol Dependence: An fMRI Study
Source: PLoS One. 2014 Jan 6;9(1):e84560. doi: 10.1371/journal.pone.0084560 (PMC3882248; doi:10.1371/journal.pone.0084560)
Supplement: Table S1 — Main effects of Alcohol>Neutral pictures. (DOCX) [file pone.0084560.s002.docx]

**Table S1. Main effects of Alcohol>Neutral pictures, *P*<.005, voxel threshold 15.**

|  |  |  |  |  |  |  |  |  |
| --- | --- | --- | --- | --- | --- | --- | --- | --- |
|  | **Area** | **BA** | **Side** | **clustersize** | **Z** | **x** | **y** | **z** |
| **Whole sample** |  |  |  |  |  |  |  |  |
|  | Inferior Occipital Gyrus |  | L | 2343 | >8 | -39 | -87 | -10 |
|  |  |  | R | 1573 | 7.69 | 39 | -87 | -10 |
|  | Superior Parietal Gyrus |  | L | 1003 | 5.56 | -34 | -61 | 50 |
|  |  | 7 | L |  | 4.8 | -32 | -71 | 50 |
|  |  |  | R | 496 | 5.33 | 28 | -59 | 48 |
|  | Posterior Cingulate Cortex | 31 | R | 1207 | 5.06 | 3 | -45 | 25 |
|  | Precuneus |  | L/R |  | 4.87 | 0 | -61 | 30 |
|  | Medial Frontal Gyrus |  | L | 491 | 4.96 | -11 | 65 | 23 |
|  |  | 8 | L |  | 4.23 | -11 | 49 | 45 |
|  |  |  | R |  | 4.04 | 5 | 56 | 30 |
|  | Inferior Frontal Gyrus |  | L | 1001 | 4.81 | -37 | 5 | 28 |
|  | Superior Frontal Gyrus | 8 | L |  | 4.01 | -39 | 17 | 55 |
|  |  |  | R | 102 | 4.64 | 14 | 37 | 55 |
|  | Globus Pallidus |  | R | 46 | 4.34 | 14 | 3 | 0 |
|  | Middle Temporal Gyrus |  | L | 136 | 4.08 | -64 | -27 | -10 |
|  | Superior Temporal Gyrus |  | R | 97 | 3.94 | 55 | -61 | 23 |
|  | Anterior Cingulate Cortex | 32 | L | 68 | 3.8 | -11 | 10 | 50 |
|  | Insula |  | L | 45 | 3.56 | -30 | 21 | -15 |
| **Alcohol Dependents** |  |  |  |  |  |  |  |  |
|  | Inferior Occipital Gyrus | 19 | L | 3040 | >8 | -39 | -84 | -13 |
|  |  | 18 | R | 1651 | 6.74 | 35 | -87 | -10 |
|  | Posterior Cingulate Gyrus |  | R | 662 | 4.58 | 5 | -45 | 25 |
|  |  |  | L |  | 4.45 | -9 | -50 | 28 |
|  | Precuneus |  | L |  | 2.6 | -14 | -59 | 38 |
|  | Insula |  | L | 709 | 4.28 | -37 | 21 | -15 |
|  | Inferior Frontal Gyrus |  | L |  | 4.49 | -34 | 10 | 28 |
|  |  |  | R | 61 | 3.68 | 44 | 26 | 23 |
|  | Inferior Parietal Lobule |  | R | 372 | 4.2 | 30 | -55 | 45 |
|  |  | 40 | R |  | 3.41 | 35 | -55 | 58 |
|  | Medial Frontal Gyrus |  | L | 124 | 3.78 | -11 | 65 | 23 |
|  |  |  | L/R |  | 3.2 | 0 | 54 | 43 |
|  |  | 9 | R |  | 3.03 | 3 | 58 | 28 |
|  | Medial Frontal Gyrus |  | L | 251 | 3.39 | -11 | 51 | 18 |
|  |  | 10 | L |  | 3.38 | -4 | 60 | -3 |
|  |  | 9 | L |  | 3.37 | -11 | 47 | 30 |
|  | Superior Frontal Gyrus | 8 | L | 33 | 3.41 | -18 | 47 | 43 |
|  | Thalamus |  | R | 31 | 3.35 | 9 | -11 | 18 |
|  | Supplementary Motor Area |  | L | 90 | 3.29 | -7 | 14 | 58 |
|  |  | 6 | R |  | 3.14 | 3 | 12 | 60 |
|  |  | 6 | L |  | 2.81 | -7 | 5 | 58 |
|  | Precentral Gyrus |  | R | 27 | 3.12 | 28 | -20 | 53 |
|  | Middle Temporal Gyrus |  | R | 22 | 3.08 | 48 | -57 | 18 |
|  |  |  | L | 15 | 3.06 | -62 | -13 | -15 |
|  | Anterior Cingulate Gyrus |  | R | 15 | 2.92 | 16 | 44 | 20 |
|  |  |  |  |  |  |  |  |  |
| **Depression/Anxiety patients** |  |  |  |  |  |  |  |  |
|  | Inferior Occipital Gyrus |  | L | 991 | 6.63 | -39 | -87 | -10 |
|  |  |  | R | 310 | 4.86 | 42 | -87 | -8 |
|  | Superior Frontal Gyrus | 10 | L | 81 | 4.23 | -23 | 65 | 15 |
|  | Middle Frontal Gyrus |  | L |  | 3.44 | -39 | 58 | 5 |
|  |  | 10 | L |  | 3.42 | -32 | 63 | 3 |
|  | Insula |  | L | 21 | 3.79 | -25 | 21 | -8 |
|  | Pallidum |  | R | 38 | 3.67 | 14 | 5 | 0 |
|  | Inferior Parietal Lobule | 40 | L | 42 | 3.5 | -37 | -57 | 53 |
|  | Posterior Cingulate Gyrus | 23 | L | 38 | 3.22 | -2 | -34 | 30 |
|  | Precuneus |  | L/R | 59 | 3.13 | 0 | -61 | 30 |
|  |  |  |  |  |  |  |  |  |
| **Healthy Controls** |  |  |  |  |  |  |  |  |
|  | Inferior Occipital Gyrus |  | L | 1248 | 5.93 | -39 | -87 | -10 |
|  |  |  | R | 743 | 5.39 | 35 | -89 | -3 |
|  |  | 18 | R |  | 3.94 | 21 | -98 | 8 |
|  | Inferior Parietal Lobule |  | L | 219 | 4.46 | -34 | -61 | 48 |
|  | Superior Frontal Gyrus | 8 | R | 105 | 4.24 | 14 | 37 | 53 |
|  |  | 8 | L | 104 | 3.84 | -11 | 49 | 45 |
|  | Supplementary Motor Area | 6 | L | 44 | 4.03 | -9 | 8 | 53 |
|  | Precuneus | 7 | R | 190 | 4.01 | 28 | -59 | 50 |
|  | Superior Parietal Gyrus |  | R |  | 3.68 | 32 | -68 | 45 |
|  | Middle Frontal Gyrus |  | L | 506 | 3.75 | -43 | 21 | 30 |
|  | Inferior Frontal Gyrus |  | L |  | 3.64 | -37 | 5 | 28 |
|  | Superior Temporal Gyrus | 39 | R | 55 | 3.65 | 53 | -61 | 23 |
|  | Precentral Gyrus | 4 | L | 47 | 3.62 | -53 | -9 | 45 |
|  | Posterior Cingulate Gyrus |  | R | 281 | 3.5 | 5 | -25 | 28 |
|  |  |  | L |  | 3.43 | 0 | -41 | 30 |
|  |  | 31 | R |  | 3.25 | 3 | -59 | 23 |
|  |  | 24 | R | 16 | 3.37 | 19 | -18 | 43 |
|  | Medial Frontal Gyrus | 9 | R | 18 | 3.37 | 9 | 60 | 33 |
|  | Middle Temporal Gyrus |  | L | 40 | 3.25 | -46 | -64 | 28 |
|  |  |  | L | 35 | 3.24 | -50 | -41 | -3 |
|  |  |  | R | 15 | 3.04 | 67 | -43 | -8 |

Abbreviations: BA, Brodmann’s Area; L, Left; R, Right; Z, Z-value; x,y,z, MNI-coordinates
