# Supplementary material for: Cue Reactivity Is Associated with Duration and Severity of Alcohol Dependence: An fMRI Study
Source: PLoS One. 2014 Jan 6;9(1):e84560. doi: 10.1371/journal.pone.0084560 (PMC3882248; doi:10.1371/journal.pone.0084560)
Supplement: Table S2 — Group comparisons. Activated areas during alcohol-cue reactivity. (DOCX) [file pone.0084560.s003.docx]

**Table S2. Group comparisons. Activated areas during alcohol-cue reactivity, P<.005 whole-brain uncorrected, clustersize threshold >= 15**

|  | **Area** | **BA** | **Side** | **clustersize** | **Z** | **x** | **y** | **z** |  |
| --- | --- | --- | --- | --- | --- | --- | --- | --- | --- |
| *Alcohol Dependents > Healthy Controls* |  |  |  |  |  |  |  |  |  |
|  | Lingual Gyrus |  | L | 44 | 3.68 | -27 | -48 | -5 |  |
|  | Anterior Cingulate Cortex |  | R | 68 | 3.43 | 16 | 10 | 33 |  |
|  |  | 32 | R | 70 | 3.51 | 7 | 35 | 15 |  |
|  | Medial Prefrontal Cortex |  | R |  | 3.33 | 19 | 42 | 20 |  |
|  |  |  | L | 16 | 3.08 | -11 | 49 | 18 |  |
|  | Inferior Frontal Gyrus |  | L | 103 | 3.49 | -37 | 35 | 20 |  |
|  | Orbitofrontal Gyrus |  | L | 20 | 3.28 | -20 | 19 | -15 |  |
| *Healthy Controls > Alcohol Dependents* |  |  |  |  |  |  |  |  |  |
|  | Middle Frontal Gyrus |  | L | 35 | 3.14 | -27 | 19 | 38 |  |
|  |  |  |  |  |  |  |  |  |  |
| *Alcohol Dependents > Depression / Anxiety Patients* |  |  |  |  |  |  |  |  |  |
|  | Caudate Nucleus |  | R | 67 | 4 | 9 | -9 | 20 |  |
|  | Precentral Gyrus |  | L | 40 | 3.87 | -41 | -9 | 43 |  |
|  | Inferior Frontal Gyrus |  | L | 151 | 3.81 | -46 | 33 | 5 |  |
|  | Orbitofrontal Gyrus | 47 | L |  | 3.15 | -32 | 33 | -13 |  |
|  |  |  | R | 16 | 3.42 | 32 | 33 | -10 |  |
|  | Posterior Cingulate Cortex | 24 | L | 43 | 3.42 | -11 | -20 | 43 |  |
|  |  | 31 | L |  | 3.22 | -2 | -18 | 45 |  |
|  | Supplementary Motor Area |  | L | 61 | 3.34 | -7 | 21 | 48 |  |
|  | Middle Occipital Gyrus |  | L | 27 | 3.26 | -37 | -82 | -3 |  |
|  | Ventral Striatum |  | L | 19 | 3.26 | -14 | 14 | -15 | * |
|  | Thalamus |  | L | 29 | 3.25 | -2 | -11 | 3 |  |
|  |  |  | R | 62 | 3.46 | 16 | -6 | 3 |  |
|  | Globus Pallidus |  | R |  | 2.7 | 23 | -11 | 0 |  |
|  | Fusiform Gyrus |  | R | 16 | 3.15 | 30 | -41 | -18 |  |
|  | Ventromedial Prefrontal Cortex | 10 | R | 46 | 3.12 | 5 | 47 | -8 |  |
|  |  | 10 | L |  | 3.04 | -2 | 51 | -8 |  |
|  | Anterior Cingulate Gyrus |  | R |  | 2.93 | 12 | 40 | -5 |  |
|  |  | 32 | R | 17 | 2.94 | 12 | 10 | 40 |  |
|  | Lingual Gyrus |  | R | 26 | 2.86 | 16 | -82 | -13 |  |
| *Depression / Anxiety Patients > Alcohol Dependents* |  |  |  |  |  |  |  |  |  |
|  | Superior Frontal Gyrus | 10 | L | 17 | 3.3 | -27 | 63 | 13 |  |
|  |  |  |  |  |  |  |  |  |  |
| *Alcohol Dependents > (Depression / Anxiety Patients & Healthy Controls)* |  |  |  |  |  |  |  |  |  |
|  | Inferior Frontal Gyrus |  | L | 199 | 3.81 | -46 | 33 | 5 |  |
|  | Orbitofrontal Cortex | 11/47 | L |  | 3.45 | -25 | 28 | -13 |  |
|  | Ventral Striatum |  | L |  | 3.44 | -14 | 14 | -15 |  |
|  |  |  | L |  | 3.28 | -9 | 14 | -13 | ** |
|  | Anterior Cingulate Cortex |  | R | 101 | 3.52 | 19 | 10 | 33 |  |
|  | Lingual Gyrus |  | L | 37 | 3.5 | -25 | -48 | -5 |  |
|  | Middle Occipital Gyrus |  | R | 67 | 3.12 | 51 | -64 | -8 |  |
|  |  |  | L | 34 | 3.07 | -37 | -78 | -3 |  |
|  | Ventromedial prefrontal cortex | 10 | R | 39 | 3.08 | 5 | 47 | -8 |  |
|  | Supplementary Motor Area |  | R | 18 | 2.99 | 7 | 3 | 60 |  |
| *(Depression / Anxiety Patients & Healthy Controls) > Alcohol Dependents* |  |  |  |  |  |  |  |  |  |
|  | No significant clusters |  |  |  |  |  |  |  |  |

Abbreviations: BA, Brodmann’s Area; L, Left; R, Right; Z, Z-value; x,y,z, MNI-coordinates

* Subthreshold FWE corrected (.05<P<.1)

** FWE corrected (P<.05)
