# Supplementary material for: Cue Reactivity Is Associated with Duration and Severity of Alcohol Dependence: An fMRI Study
Source: PLoS One. 2014 Jan 6;9(1):e84560. doi: 10.1371/journal.pone.0084560 (PMC3882248; doi:10.1371/journal.pone.0084560)
Supplement: Table S3 — Regression analyses during alcohol-cue reactivity within the group of Alcohol Dependent patients. (DOCX) [file pone.0084560.s004.docx]

**Table S3. Regression analyses during alcohol-cue reactivity within the group of Alcohol Dependent patients, P<.005 whole-brain uncorrected, clustersize threshold = 18.**

|  |  | **Area** | **BA** | **Side** | **clustersize** | **Z** | **x** | **y** | **z** |  |
| --- | --- | --- | --- | --- | --- | --- | --- | --- | --- | --- |
| **Severity** |  |  |  |  |  |  |  |  |  |  |
|  | **Positive** |  |  |  |  |  |  |  |  |  |
|  |  | Superior Frontal Gyrus |  | L | 47 | 3.87 | -20 | 35 | 50 |  |
|  |  |  |  | R | 59 | 3.31 | 16 | 37 | 45 |  |
|  |  | Inferior Frontal Gyrus |  | L | 27 | 3.38 | -41 | 21 | 15 |  |
|  |  | Anterior Cingulate Cortex |  | R | 18 | 3.24 | 12 | 24 | 38 |  |
|  |  | Thalamus |  | L | 19 | 3.11 | -14 | -20 | 15 |  |
|  | **Negative** |  |  |  |  |  |  |  |  |  |
|  |  | Anterior Putamen |  | R | 63 | 3.79 | 25 | 21 | -8 | ** |
|  |  | Anterior Cingulate Cortex | 32 | L | 19 | 3.26 | -9 | 33 | -8 |  |
| **Duration** |  |  |  |  |  |  |  |  |  |  |
|  | **Positive** |  |  |  |  |  |  |  |  |  |
|  |  | Middle Temporal Gyrus |  | L | 59 | 4.52 | -48 | -45 | 3 |  |
|  |  | Anterior Cingulate Cortex |  | R | 129 | 4.1 | 16 | 21 | 43 |  |
|  |  | Superior Frontal Gyrus | 8 | L | 108 | 4.01 | -20 | 31 | 53 |  |
|  |  |  |  | R | 37 | 3.51 | 12 | 28 | 58 |  |
|  |  | Middle Frontal Gyrus | 6 | R | 24 | 3.03 | 44 | 10 | 55 |  |
|  |  |  | 9 | R | 50 | 3.47 | 37 | 33 | 40 |  |
|  |  | Posterior Insula | 13 | L | 39 | 3.93 | -32 | -29 | 13 |  |
|  |  | Angular Gyrus |  | R | 100 | 3.91 | 44 | -57 | 33 |  |
|  |  | Postcentral Gyrus |  | L | 54 | 3.67 | -23 | -20 | 58 |  |
|  |  |  | 3 | L | 108 | 3.65 | -50 | -18 | 45 |  |
|  |  |  |  | R | 21 | 3.42 | 32 | -27 | 43 |  |
|  |  |  | 1 | R | 30 | 3.24 | 55 | -20 | 45 |  |
|  |  | Posterior Putamen |  | L | 67 | 3.64 | -30 | -2 | -3 | ** |
|  |  | Middle Cingulate Cortex |  | R | 84 | 3.36 | 16 | -4 | 43 |  |
|  |  | Precuneus |  | L | 39 | 3.32 | -18 | -64 | 30 |  |
|  |  | Supplementary Motor Area | 6 | L | 42 | 3.22 | -4 | -27 | 53 |  |
|  |  | Middle Occipital Gyrus | 39 | L | 22 | 3.14 | -41 | -73 | 33 |  |
|  | **Negative** |  |  |  |  |  |  |  |  |  |
|  |  | Middle Occipital Gyrus |  | R | 109 | 3.8 | 30 | -94 | 10 |  |
|  |  |  |  | L | 18 | 3.1 | -16 | -98 | 8 |  |
| **Self-reported cue-induced craving** |  |  |  |  |  |  |  |  |  |  |
|  | **Positive** |  |  |  |  |  |  |  |  |  |
|  |  | Inferior Occipital Gyrus |  | L | 16 | 3.86 | -37 | -91 | -8 |  |
|  |  | Parahippocampal Gyrus |  | R | 27 | 3.17 | 44 | -18 | -18 |  |
|  | **Negative** |  |  |  |  |  |  |  |  |  |
|  |  | Superior Frontal Gyrus |  | R | 44 | 4.14 | 32 | 8 | 40 |  |
|  |  | Anterior Cingulate Gyrus |  | L | 46 | 3.43 | -7 | 26 | -5 |  |
|  |  |  | 24 | R |  | 3.22 | 3 | 21 | -5 |  |
|  |  | Fusiform Gyrus |  | R | 25 | 3.02 | 28 | -71 | -10 |  |
|  |  |  | 37 | L | 16 | 2.98 | -34 | -43 | -13 |  |
| **Depression symptoms (IDS)** |  |  |  |  |  |  |  |  |  |  |
|  | **Positive** |  |  |  |  |  |  |  |  |  |
|  |  | Postcentral Gyrus |  | R | 105 | 3.86 | 46 | -27 | 33 |  |
|  |  |  | 4 | R |  | 3.47 | 58 | -20 | 38 |  |
|  |  |  | 40 | R |  | 2.94 | 51 | -36 | 38 |  |
|  |  |  |  | L | 15 | 3.03 | -50 | -13 | 25 |  |
|  | **Negative** |  |  |  |  |  |  |  |  |  |
|  |  | Precuneus | 19 | L | 46 | 3.87 | -39 | -73 | 40 |  |
|  |  | Inferior Parietal Lobule | 40 | L |  | 2.66 | -48 | -64 | 43 |  |
|  |  |  | 40 | R | 75 | 3.63 | 51 | -59 | 43 |  |
|  |  | Supplementary Motor Area | 6 | L | 34 | 3.32 | -16 | 19 | 60 |  |
|  |  |  |  | L |  | 2.78 | -11 | 8 | 63 |  |
| **Anxiety symptoms (BAI)** |  |  |  |  |  |  |  |  |  |  |
|  | **Positive** |  |  |  |  |  |  |  |  |  |
|  |  | No significant clusters |  |  |  |  |  |  |  |  |
|  | **Negative** |  |  |  |  |  |  |  |  |  |
|  |  | IFG | 45 | R | 34 | 3.66 | 42 | 19 | 3 |  |
|  |  |  |  | R | 42 | 3.54 | 35 | 26 | 20 |  |
|  |  | Insula | 47 | L | 50 | 3.28 | -37 | 21 | 0 |  |
|  |  | Precuneus | 31 | L | 17 | 3.27 | -9 | -61 | 25 |  |
|  |  | Inferior Parietal Lobule | 40 | R | 26 | 3.2 | 44 | -59 | 48 |  |
|  |  |  | 19 | L | 42 | 3.1 | -32 | -68 | 40 |  |
|  |  | Superior Temporal Gyrus |  | L | 37 | 3.06 | -62 | -29 | 10 |  |
|  |  | Supplementary Motor Area |  | R | 21 | 3.03 | 3 | -2 | 50 |  |
|  |  |  | 6 | L |  | 2.77 | -2 | -9 | 55 |  |
|  |  |  | 6 | R | 18 | 3.01 | 3 | -25 | 53 |  |
| **Smoking** |  |  |  |  |  |  |  |  |  |  |
|  | **Positive** |  |  |  |  |  |  |  |  |  |
|  |  | Superior Temporal Gyrus |  | L | 24 | 3.83 | -39 | 10 | -20 |  |
|  |  | Anterior Cingulate / White matter |  | L | 28 | 3.28 | -14 | 35 | 15 |  |
|  |  | Inferior Parietal Lobule | 40 | L | 17 | 3.24 | -43 | -48 | 40 |  |
|  | **Negative** |  |  |  |  |  |  |  |  |  |
|  |  | Cuneus |  | R | 69 | 3.66 | 25 | -82 | 8 |  |
|  |  | Precentral Gyrus |  | L | 24 | 3.36 | -34 | -6 | 40 |  |
|  |  | Inferior Occipital Gyrus |  | L | 50 | 3.21 | -25 | -94 | -10 |  |
|  |  |  |  | L |  | 3.11 | -20 | -89 | -5 |  |
|  |  | Middle Temporal Gyrus |  | R | 35 | 3.14 | 51 | -13 | -15 |  |
|  |  | Superior Temporal Gyrus |  | R |  | 3.04 | 51 | -13 | -5 |  |
|  |  |  |  |  |  |  |  |  |  |  |

Abbreviations: BA, Brodmann’s Area; L, Left; R, Right; Z, Z-value; x,y,z, MNI-coordinates

** FWE corrected (P<.05)
